# Cognitive reactivity compared to other risk factors in the prediction of depressive episodes over two and nine years: a longitudinal cohort study

**DOI:** 10.1080/13651501.2025.2476509

**Published:** 2025-03-16

**Authors:** Ericka C. Solis, Ingrid V. E. Carlier, Robert A. Schoevers, Brenda W. J. H. Penninx, Albert M. van Hemert, A. J. Willem van der Does

**Affiliations:** a Department of Psychiatry, Leiden University Medical Center, Leiden, The Netherlands; b Department of Psychiatry, University of Groningen, Groningen, The Netherlands; c Department of Psychiatry and Amsterdam Public Health Research Institute, University Medical Center Amsterdam, The Netherlands; d Department of Clinical Psychology, Leiden University, Leiden, The Netherlands

**Keywords:** cognitive vulnerability, risk factors, recurrent depression, depression onset, longitudinal study

## Abstract

**Objective:**

Cognitive Reactivity (CR) is the (re-)activation of negative cognitions by dysphoric mood. We examined whether CR predicts depressive episodes across 2 and 9 years, beyond subclinical depressive symptoms, neuroticism, and previous depressive episodes.

**Methods:**

Participants (*N* = 1,734) from the Netherlands Study of Depression and Anxiety (NESDA) were never-depressed or remitted-depressed for ≥1 month prior to baseline. We examined 2-year and 9-year predictions using Cox’s survival analysis and logistic regression, respectively. Two-year coefficient-based weight-points were calculated and evaluated using ROC analysis.

**Results:**

CR was a statistically-significant predictor of two-year depressive episodes, with an odds ratio of 1.04, 95% CI (1.02–1.06), and over nine years, with an adjusted hazard ratio of 1.01, 95% CI (1.01–1.02). The influence of CR and subclinical depressive symptoms decreased as the number of episodes increased, especially in ≥ 3 past episodes. Calculated weight-points correctly predicted 33.5% of participants who developed 2-year depression, compared to a 17.8% base rate (sensitivity = .81, specificity = .66).

**Conclusions:**

CR is a moderately strong predictor of depressive episodes across 2 and 9 years. In participants with ≥ 3 prior episodes, depression history is such a strong predictor that a ceiling effect occurs, removing any added value of other predictors.

## KEY POINTS

- CR is a moderately strong, independent predictor of first (onset) and second (relapse) depressive episodes across 2- and 9-years;
- After three past depressive episodes, personal history of depression is stronger than CR as a predictor of future depressive episodes, although the effect is so strong that no other factor added value, suggesting a ceiling effect;
- The 2-year risk for depression relapse is higher than 70%, with higher risks for women than men;
- Our findings confirm the importance of early treatment and relapse prevention in clinical treatment for patients with a depression history.

## Introduction

Depression is a common and often recurrent disorder (Solis et al., 2021). Several vulnerability factors, or predictors, of recurrence exist. Well-established predictors include subclinical depressive symptoms and past depressive episodes. Individuals with subclinical depressive symptoms (SDS) face relapse sooner than those without symptoms (Judd et al., 1998). Similarly, those with past depressive episodes have a higher risk for relapse: experiencing one past episode is associated with a 40–60% relapse risk, while two past episodes is associated with a 70% risk, and three past episodes with a 90% risk (Eaton et al., 2008; Solomon et al., 2000). Additionally, each relapse carries a 10–20% risk of becoming unremitting and chronic (Judd et al., 2000; Lee, 2003). If we can identify individuals at high risk for depression, early interventions that may prevent depression onset or relapse can be offered (Beshai et al., 2011; Lutz et al., 2009).

One promising predictor of relapse is cognitive reactivity (CR) (Figueroa et al., 2015; Scher et al., 2005; Segal et al., 2006). CR has also been shown to predict first depression onset in a population at increased risk (Kruijt et al., 2013). CR is the mood-related (re-)activation of negative cognitions, which are thought to underlie the vulnerability to developing and maintaining depression (Alloy et al., 1999, 2006; Beck, 1976; Jarrett et al., 2012; Otto et al., 2007). These negative cognitions (e.g., ‘I’m worthless’) may be developed in childhood and are presumed to be inactive prior to the onset of depression or during remission of depression (Kruijt et al., 2013; Teasdale, 1988). In line with the diathesis-stress model, sad mood states, even when mild, may (re-)activate these negative cognitions (Hollon et al., 1987; Ingram & Ritter, 2000; Miranda et al., 1998). Once activated, these negative cognitions may further trigger a negative memory bias and a pattern of negative information processing, leading to depression (Scher et al., 2005). Previous research found that lower CR in individuals after mindfulness-based cognitive therapy was associated with a reduction in depressive symptoms (Cladder-Micus et al., 2018), suggesting that the relation between CR and depression relapse may be causal. However, research on CR has been limited to the prediction of depression using follow-up periods less than 3.5 years (Figueroa et al., 2015; Kruijt et al., 2013; Segal et al., 2006).

Another predictor for depression onset and relapse is the personality trait, neuroticism (Kotov et al., 2010; Renner et al., 2013; Struijs et al., 2018; van Eeden et al., 2019; Zuroff et al., 2004). Neuroticism is the propensity to experience intense negative emotions. Underlying this emotional instability is a negative cognitive-related component: perceiving the self as incapable and the world as threatening (Barlow et al., 2014; Costa & McCrae, 1992; Hankin et al., 2007; Lahey, 2009). CR and neuroticism have been shown to be partially overlapping, yet distinct concepts (Hankin et al., 2007; Solis et al., 2017; Zuroff et al., 2004). While CR refers to a lowered threshold to (re-)activating negative cognition during sad mood, neuroticism refers to a lowered threshold to a negative mood during daily activities and hassles. In sum, individuals with higher neuroticism scores experience more emotional dysregulation, which may re-activate negative thoughts and schemas, particularly in those with high CR scores (Barnhofer & Chittka, 2010).

It is not yet clear whether CR remains a predictor of depression for longer follow-up periods than 3.5 years (Figueroa et al., 2015), beyond the effect of other known vulnerability factors of depression relapse, such as neuroticism, SDS, and past depressive episodes. In the present study, we aimed to investigate the ability of CR to predict depressive episodes in never-depressed individuals or remitted-depressed individuals over a 2- and 9-year period. We expected that higher CR scores would predict a depressive episode, adjusting for sociodemographic and vulnerability factors.

## Materials and method

### Data source and participants

Data were extracted from the Netherlands Study of Depression and Anxiety (NESDA, www.nesda.nl), an ongoing naturalistic cohort study designed to investigate the course and consequences of depression and anxiety (for the extensive NESDA protocol, see Penninx et al. (NESDA Research Consortium, 2008)). At baseline, participants, aged 18 to 65, with or without a lifetime depressive and/or anxiety disorder were included (*N* = 2,981). After baseline, follow-up assessments were conducted at 1 year (*n* = 2,445; 82.0%; questionnaires only), 2 years (*n* = 2,596; 87.1%), 4 years (*n* = 2,402; 80.8%), 6 years (*n* = 2,256; 75.7%), and 9 years (*n* = 2,069; 69.4%). Exclusion criteria included the clinically overt presence of psychotic, obsessive compulsive, bipolar or severe substance-use disorders, and non-fluency in Dutch. Ethical approval was granted by the ethical committees of participating universities and medical centres.

### Study sample

Derived from the NESDA cohort, we included individuals without depression (i.e., never-depressed or remitted-depressed) ≥ 1 month prior to baseline, with complete baseline data, and at least one follow-up assessment. To explore the effect of depression history and make our results comparable to prior studies, we created two subsamples based on depression history (i.e., 0 or ≥1 past episode prior to baseline).

### Measures

#### Depressive disorders

The presence of a depressive episode (major depressive disorder (MDD) or dysthymic disorder (DD)) was our main outcome of interest. It was assessed with the Composite International Diagnostic Interview (CIDI, version 2.1 (Wittchen, 1994; Wittchen & Nelson, 1996)), which is a standardised diagnostic interview used to classify DSM-IV psychiatric diagnoses. It has been shown to have high interrater reliability, high test-retest reliability, and high validity for depressive and anxiety disorders (Andrews & Peters, 1998; Haro et al., 2006; Romera et al., 2002; Wittchen, 1994; Wittchen et al., 1991). In NESDA, participants were assessed for depressive disorders at baseline and at the 2-, 4-, 6-, and 9-year assessments, with the recency of the past 12-, 6-, and 1-month(s). To select our sample for the current study, the 1-month recency at baseline was used. For the outcome, the 12-month recency was used, giving the widest possible detection period for occurrence of depression in this study.

#### Cognitive reactivity (CR)

CR was our primary predictor of interest. It was measured with the Leiden Index of Depression Sensitivity, a self-report questionnaire with five subscales. Participants rated the extent to which each statement applied to them on a five-point Likert scale (0 = *not at all*; 1 = *a bit*; 2 = *moderately*; 3 = *strongly*; and 4 = *very strongly*). The total score was obtained by summing item responses of all subscales. The first and second revision of the LEIDS (i.e., LEIDS-R and LEIDS-RR) showed acceptable/good criterion and divergent validity and moderate-to-high internal consistency (Solis et al., 2017). In NESDA, CR was measured with this earlier 34-item version of the questionnaire (first revision; LEIDS-R) at baseline and the 2-year assessment (Van der Does and Williams, 2013). For the current study, however, we recalculated CR at baseline for the 30-item second version of the questionnaire (LEIDS-RR), which has improved psychometrics data (Solis et al., 2017).

#### Neuroticism

Neuroticism was considered a relevant baseline predictor and potential overlapping construct with CR. It was measured using the NEO Five Factor Inventory (NEO-FFI), a 60-item self-report questionnaire that measures the Big Five personality domains, including neuroticism. Each personality domain consists of 12 items (Costa & McCrae, 1992). The items (e.g., ‘I am not a worrier’) are rated on a five-point Likert scale (1 = *strongly disagree* to 5 = *strongly agree*). For the current study, we used the neuroticism score at baseline. Neuroticism scores have shown adequate internal consistency and good reliability (Costa & McCrae, 1992; Robins et al., 2001).

#### Subclinical depressive symptoms (SDS)

SDS level was considered a relevant baseline predictor and potential confounder. It was measured using the Inventory of Depressive Symptomatology, Self-Report (IDS-SR), a 28-item questionnaire used to measure four-point Likert scale (0 to 3) (Rush et al., 1986, 1996; Trivedi et al., 2004) and a total score ranging from 0–84. The cut-off score for mild depression is 14 (Nolen & Dingemans, 2004; Rush et al., 1996). The IDS-SR has adequate internal consistency, test-retest stability, as well as adequate convergent and discriminant validity (Rush et al., 1996).

#### Depression history

Depression history refers to the number of past depressive episodes (PDE), as reported on the CIDI at baseline. For the current study, the continuous variable was categorised as no (0), few (1–2), or many (≥ 3) PDE.

#### Age, sex, and level of education

These were included as relevant baseline predictors and potential confounders. For the current study, we calculated age using the participant’s birthdate. For sex, we used a binary variable (female/male). For education level, we used the number of years in school.

### Statistical analysis

All analyses were conducted using IBM SPSS statistical software (version 25, SPSS Inc., Chicago, Illinois). Descriptive statistics were used for baseline characteristics. To examine whether baseline CR predicts depression over a longer longitudinal period of 9 years, we conducted multivariate Cox’s proportional hazards analyses with the time to the first depressive episode (MDD or DD) as the main outcome, using the 2-, 4-, 6-, and 9-year follow-up assessments. Participants lost to follow-up or without a depressive episode were censored. A *base* model with CR as a predictor and an *adjusted* model incorporating age, sex, education, neuroticism, SDS, and PDE were estimated. Adjusted models were also estimated for subgroups with and without prior depression to compare results with previous literature.

To evaluate the predictive strength of CR over 2 years compared to other vulnerability factors, we conducted logistic regression with 2-year depressive episode as the main outcome. Factors with *p* > .10 from the Cox analysis were included. Interaction terms with PDE were examined and retained if *p* > .05. Nine-year logistic regression models were estimated for comparison.

To allow for meaningful clinical interpretation, questionnaire scores of our predictors were converted into weight-points using the procedure described by Sullivan et al. (2004). Raw questionnaire scores were first grouped into categories (e.g., 25–34; 35–44) and assigned representative values (e.g., 25–34 = 30; 35–44 = 40). These values were multiplied using their respective beta coefficient from the logistic regression model, then divided by the beta of CR, selected as the constant. The constant represented the number of regression units corresponding to one point. We conducted ROC analysis to evaluate the ability of these weight-points to identify a high 2-year depression risk (Carter et al., 2016). Baseline risk estimate profiles for men and women were calculated using their mean baseline scores and total weight-points (Sullivan et al., 2004). Nine-year profiles were estimated for comparison. For details regarding these analyses, see Supplementary Material.

## Results

### Sample selection

Of the 2,981 participants, 852 (28.6%) were currently depressed at baseline and thus excluded. Of the remaining 2,129 participants, 299 had missing data on the following baseline measures: 212 (10.0%) regarding cognitive reactivity; 21 (1.0%) regarding subclinical depressive symptoms; 90 (4.2%) regarding the number of previous depressive episodes; and 20 (0.9%) regarding neuroticism. Additionally, 96 (5.3%) participants dropped out after the baseline assessment and lacked CIDI follow-up information. This resulted in a sample of 1,734 never-depressed or remitted-depressed individuals, with complete baseline data and at least one of the 2-, 4-, 6-, and 9-year follow-up assessments. Moreover, for the logistic regression assessing 2-year depression risk, there were *n* = 45 participants missing the 2-year depression outcome, resulting in a total sample of 1,689 participants for the 2-year analyses. For the logistic regression assessing 9-year depression risk, there were *n* = 345 participants missing the 9-year depression outcome, resulting in a total sample of 1,389 participants for the 9-year analyses. Considering the total sample, excluded participants (i.e., with current depression) differed significantly from the included participants on the following variables: setting (χ_(2)_ = 47.3 *p* < .0001), years of education (*t*(604) = 3.9, *p* < .0001), baseline neuroticism score (*t*(2107) = −2.5, *p* = .013), and baseline depressive symptoms (*t*(2106) = −4.4, *p* < .0001).

### Demographic and clinical characteristics

Baseline demographic and clinical characteristics of the total sample and the subsamples are presented in Table 1. At baseline, participants were on average 41.9 years old (*SD* = 13.5), with an overrepresentation of females (67.5%). About half (*n* = 891; 51.4%) had no PDE and 843 (48.6%) experienced ≥1 PDEs. Of these, 551 (31.8%) had 1 or 2 PDEs, and 292 (16.8%) had ≥3 PDEs. The average IDS-SR score of participants with a depression history was higher than 14, which reflected having at least mild depressive symptoms, despite no longer meeting depression disorder criteria at baseline. Participants with a depression history had statistically significant higher baseline CR scores, *t*(1732) = −17.45, *p* < .001, neuroticism scores, *t*(1732) = −17.68, *p* < .001, and SDS scores, *t*(1732) = −15.58, *p* < .001 than participants without a depression history.

**Table 1.** Baseline demographic and clinical characteristics of participants with and without a depression history at baseline.^a^

Sociodemographic or clinical variables	Total (*N* = 1734)	Subsample 0 episodes (*n* = 891)	Subsample 1+ episodes (*n* = 843)	Subsample 1–2 episodes (*n* = 551)	Subsample 3+ episodes (*n* = 292)
Age, mean (*SD*)	41.9 (13.5)	41.7 (14.3)	42.2 (12.6)	42.2 (13.0)	42.1 (11.9)
Gender, female, *N* (%)	1171 (67.5)	566 (63.5)	605 (71.8)	387 (70.2)	218 (74.7)
Education years, mean (*SD*)	12.6 (3.2)	12.7 (3.3)	12.5 (3.2)	12.4 (3.2)	12.8 (3.1)
Cognitive reactivity, LEIDS-RR, mean (*SD*)	24.5 (15.7)	18.6 (13.7)**	30.7 (15.3)**	30.0 (14.9)**	34.1 (15.4)**
Neuroticism, NEO-FFI, mean (*SD*)	33.3 (8.9)	29.9 (8.4)**	36.9 (8.0)**	35.2 (8.1)**	38.8 (7.5)**
Subclinical depressive symptoms, IDS-SR, mean (*SD*)	15.4 (10.7)	11.8 (9.5)**	19.2 (10.5)**	18.5 (10.5)**	20.8 (10.4)**
Past depressive episodes, CIDI, mean (*SD*)	3.7 (7.7)	0 (0.0)	3.7 (7.7)	1.2 (0.4)	8.5 (11.8)
Comorbid anxiety disorder, CIDI, *N* (%)	446 (25.7)	172 (19.3)**	274 (32.5)**	172 (31.2)**	102 (34.9)**

*Note.* Depression refers to either a major depressive disorder or dysthymic disorder. All participants did not have depression at least 1 month prior to baseline as determined using the CIDI. Participants may have a comorbid anxiety disorder at baseline.

** *p* < .001, as tested using a *t*-test (0 vs. 1+ episodes) or one-way ANOVA (0 vs. 1–2 vs. 3+ episodes); refers only to clinical variables.

^a^ Determined using the past number of episodes reported on the CIDI at baseline.

LEIDS-RR: Leiden Index of Depression Sensitivity second Revision; NEO-FFI: NEO Five Factor Inventory; IDS-SR: Inventory of Depressive Symptomatology - Self-Report; CIDI: Composite International Diagnostic Interview.

### Prediction of depressive episodes using cognitive reactivity

During the 9-year follow-up, 449 (25.9%) participants experienced at least one depressive episode. Table 2 shows the hazard ratios (HR) of the Cox’s analyses across 9-year follow-up. The base model (A) showed that CR was a statistically significant independent predictor of depression across 9 years (HR_CR/BaseA_ = 1.04, 95% CI [1.03–1.04], *p* < .001). In the adjusted model, CR remained a statistically significant predictor (HR_CR/AdjustedB_ = 1.01, 95% CI [1.01–1.02], *p* < .02). When examining the prediction of first depression onset versus depression relapse, CR was a statistically significant predictor of first depression onset (HR_CR/AdjustedD_ = 1.03, 95% CI [1.02–1.05], *p* < .0001) but not of relapse (HR_CR/AdjustedF_ = 1.01, 95% CI [1.00–1.01], *p* = .22).

**Table 2.** Prediction of depressive episodes based on cognitive reactivity across nine-years, adjusted for other relevant baseline factors, using Cox’s proportional hazards analysis.

Sample	Total (*N* = 1734)		Past depression - No (*n* = 891)		Past depression - Yes (*n* = 843)	
Outcome	Any depressive episode		First episode		Next episode - relapse	
Predictors	(A) Base: HR (95% CI), *p*-value	(B) Adjusted: HR (95% CI), *p*-value	(C) Base: HR (95% CI), *p*-value	(D) Adjusted: HR (95% CI), *p*-value	(E) Base: HR (95% CI), *p*-value	(F) Adjusted: HR (95% CI), *p*-value
Cognitive reactivity	1.04 (1.03–1.04), <.001*	1.01 (1.00–1.02), .02*	1.05 (1.04–1.06), <.001*	1.03 (1.02–1.04), <.001*	1.02 (1.01–1.03), <.001*	1.01 (1.00–1.01), .18
Age	N.I.	1.00 (.99–1.00), .32	N.I.	.99 (.97–1.00), .06^§^	N.I.	1.00 (.99–1.01), .55
Female^a^	N.I.	1.20 (.97–1.48), .10^§^	N.I.	1.73 (1.12–2.68), .01*	N.I.	1.06 (.83–1.34), .66
Education	N.I.	.98 (.95–1.01), .18	N.I.	.94 (.89–1.00), .04*	N.I.	.99 (.96–1.03), .72
Neuroticism	N.I.	1.02 (1.00–1.04), .04*	N.I.	.99 (.96–1.03), .77	N.I.	1.02 (1.00–1.04), .06^§^
SDS	N.I.	1.04 (1.02–1.05), <.001*	N.I.	1.07 (1.04–1.10), <.001*	N.I.	1.03 (1.01–1.04), .001*
1–2 past episodes^b^	N.I.	1.97 (1.55–2.51), <.001*	N.I.	N.I.	N.I.	N.I.
≥3 past episodes^b^	N.I.	2.29 (1.75–2.99), <.001*	N.I.	N.I.	N.I.	N.I.

* *p* < .05

^§^ *p* < .10.

^a’^ Male gender’ was considered the reference category.

^b’^ 0 past episodes’ was considered the reference category.

HR: Hazard ratio; CI: Confidence interval; N.I.: Not included; SDS: Subclinical depressive symptoms.

### Strength of cognitive reactivity relative to vulnerability factors

For the 2-year logistic model, CR, (female) gender, neuroticism, SDS, and depression history (PDE) met criteria for selection (see Table 2). There were 1,689 participants with complete data for inclusion in the 2-year prediction model (97.4% of total sample).

Table 3 shows the odds-ratios and regression coefficients of the logistic regression models. The interactions of depression history with CR and SDS were statistically significant (see Model 1). We re-estimated the model using only these aforementioned statistically significant interactions. In Model 2, the odds-ratio of CR on depression-risk decreased from 1.04 to 0.98 after 1–2 PDE, then 0.96 after ≥ 3 PDE (where the OR refers to each point increase on the LEIDS-RR). Similarly, the odds-ratio of SDS on depression-risk decreased from 1.06 to 0.99 for 1–2 PDE, and 0.94 for ≥ 3 PDE. Depression history, as an independent predictor, increased the odds of experiencing depression by 4 times in those with 1–2 PDE and by 24 times in those with ≥ 3 PDE (see Model 2). Compared to the 2-year model, the effect of CR and other factors on 9-year depression-risk were generally similar. However, for 9-year risk, female gender and depression history ≥3 episodes had a slightly higher OR, depression history of 1–2 episodes had a slightly lower OR, and the interaction of depression history with CR was not statistically significant (see Model 1 vs 3). Supplementary Tables 1 and 2 show the overview of calculated weight-points and participant distribution, respectively, based on regression coefficients. Calculated weight-points correctly predicted 33.5% of participants who developed 2-year depression, compared to a 17.8% base rate (sensitivity = .81, specificity = .66).

**Table 3.** Selected predictors of depressive episodes after two or nine years in participants without baseline depression using logistic regression.

	Model 1: 2-year depression		Model 2: 2-year depression*		Model 3: 9-year depression^c^		Model 3: 9-year depression*	
Predictors and interactions	OR (95% CI)	Beta *(p*-value)	OR (95% CI)	Beta (*p-*value)	OR (95% CI)	Beta (*p-*value)	OR (95% CI)	Beta (*p-*value)
CR	1.04 (1.02–1.07)	.043 (<.001)	1.04 (1.02–1.06)	.038 (<.001)	1.03 (1.00–1.06)	.030 (.04)	1.02 (1.00–1.05)	.024 (.08)
Female gender^a^	1.57 (.88–2.80)	.453 (.12)	1.35 (.99–1.84)	.297 (.06)	2.09 (.92–4.79)	.739 (.08)	1.23 (.84–1.79)	.204 (.29)
Neuroticism	1.01 (.96–1.05)	.005 (.83)	1.03 (1.00–1.06)	.029 (.02)	.98 (.92–1.04)	−0.020 (.50)	1.02 (.98–1.05)	.016 (.31)
SDS	1.07 (1.04–1.11)	.070 (<.001)	1.06 (1.03–1.09)	.060 (<.001)	1.09 (1.04–1.14)	.083 (<.001)	1.07 (1.03–1.11)	.066 (<.001)
1–2 past episodes^b^	2.46 (.43 − 13.95)	.900 (.31)	4.10 (1.74–9.67)	1.411 (<.01)	1.94 (.23–16.68)	.664 (.55)	4.48 (1.57–12.77)	1.500 (<.01)
≥3 past episodes^b^	9.87 (1.35–72.45)	2.290 (.02)	23.75 (9.15–61.67)	3.168 (<.001)	10.37 (.98–110.24)	2.339 (.05)	13.76 (4.28–44.19)	2.621 (<.001)
CR X 1–2 past episodes^b^	.97 (.95–1.00)	−0.029 (.04)	.98 (.95–1.00)	−0.023 (.07)	.99 (.95–1.02)	−0.015 (.41)	.99 (.96-.103)	−0.005 (.75)
CR X ≥ 3 past episodes^b^	.96 (.93-.98)	−0.046 (.00)	.96 (.94-.99)	−0.039 (<.01)	.97 (.94–1.01)	−0.028 (.14)	.98 (.95–1.01)	−0.022 (.20)
Gender X 1–2 past episodes^b^	.72 (.34–1.52)	−0.325 (.39)	N.I.	N.I.	.46 (.17–1.26)	−0.767 (.13)	N.I.	N.I.
Gender X ≥ 3 past episodes^b^	.95 (.41–2.22)	−0.052 (.90)	N.I.	N.I.	.56 (.19–1.63)	−0.588 (.28)	N.I.	N.I.
Neuro X 1–2 past episodes^b^	1.03 (.97 − 1.10)	.032 (.29)	N.I.	N.I.	1.06 (.99–1.15)	.060 (.12)	N.I.	N.I.
Neuro X ≥ 3 past episodes^b^	1.04 (.97 − 1.11)	.038 (.27)	N.I.	N.I.	1.04 (.95–1.13)	.034 (.43)	N.I.	N.I.
SDS X 1–2 past episodes^b^	.98 (.94–1.02)	−0.019 (.39)	.99 (.96–1.03)	−0.005 (.77)	.95 (.89− 1.00)	−0.055 (.05)	.97 (.93–1.02)	−0.029 (.20)
SDS X ≥ 3 past episodes^b^	.93 (.89− .97)	−0.074 (.00)	.94 (.91-.98)	−0.058 (<.01)	.95 (.89–1.00)	−0.056 (.06)	.96 (.92–1.01)	−0.040 (.09)

*Note.* For two-year depression risk, there were *n* = 45 participants missing the two-year depression outcome; therefore, the total sample was *N* = 1,689. The following had a depressive episode at two years: of those with 0 past episodes, 77; of those with 1–2 past episodes, 135; of those with ≥3 past episodes, 85. For nine-year depression risk, there were *n* = 345 participants missing the nine-year depression outcome; therefore, the total sample was *N* = 1,389. The following had a depressive episode at nine years: of those with 0 past episodes, 38; of those with 1–2 past episodes, 77; of those with ≥3 past episodes, 63.

* Interactions were selected in Model 2 if at least one of the categories in the interaction group in Model 1 was *p* < .05. The same interactions were selected and tested in Model 4 for 9-year depression.

^a’^ Male gender’ was considered the reference category.

^b’^ 0 past episodes’ or ‘[predictor] X 0 past episodes’ was considered the reference category.

^c^ Model 3 for nine-year depression risk was estimated as a comparison to Model 1.

OR: Odds ratio; CI: Confidence interval; CR: cognitive reactivity; Neuro: neuroticism; SDS: subclinical depressive symptoms; N.I.: not included.

Table 4 shows risk estimates for developing 2- and 9-year depression based on average baseline scores, depression history, and corresponding weight-points. Regarding 2-year depression, participants with 0 PDE (*n* = 891) had a depression-risk between .16 and .26, where SDS played the strongest role (16 points), followed by CR (13 points), then neuroticism and gender (both 8 points). In participants with 1–2 PDE (*n* = 551), the risk estimate was at least .81, where depression history played the strongest role, reducing the impact of CR (from 13 to 9 points). In participants with ≥ 3 PDE (*n* 292), the risk estimate was at least .93, where depression history (83 points) played the strongest role, followed by neuroticism (15 points), then gender (8 points). Risk estimates were higher for women than men. Factor contribution was generally similar across 2 and 9 years. However, in 9-year depression risk, CR contributed more in participants with 1–2 PDE than in 2-year results, and SDS contributed the more than CR and neuroticism in participants with ≥ 3 PDE. The latter was not confirmed when examining stratified subgroups using Cox analysis (see Supplementary Table 3).

**Table 4.** Risk estimates for men and women developing depression after two and nine years based on logistic regression coefficients, mean baseline questionnaire scores, depression history, and corresponding weight-points.

	0 Past episodes (*n* = 891)		1–2 Past episodes (*n* = 551)		≥ 3 Past episodes (*n* = 292)	
Predictor variables	Weight-points 2 yr | 9 yr	Mean score baseline	Weight-points 2 yr | 9 yr	Mean score baseline	Weight-points 2 yr | 9 yr	Mean score baseline
Gender, female	8 | 9	Female	8 | 9	Female	8 | 9	Female
Gender, male	0 | 0	Male	0 | 0	Male	0 | 0	Male
Cognitive reactivity (LEIDS-RR) at baseline	13 | 13	18.6	9 | 19	30.0	−1 | 2	34.1
Neuroticism (NEO-FFI) at baseline	8 | 7	29.9	15 | 13	35.2	15 | 13	38.8
Subclinical depressive symptoms (IDS-SR) at baseline	16 | 27	11.8	14 | 15	18.5	1 | 22	20.8
Number of past depressive episodes (CIDI) at baseline	0 | 0	0	38 | 62	1	83 | 109	8
Total weight-points, Female	45 | 56		84 | 119		106 | 158	
Risk estimates, Female	.26 | .14		.89 | .79		.96 | .97	
Total weight-points, Male	37 | 47		76 | 110		98 | 149	
Risk estimates, Male	.16 | .10		.81 | .71		.93 | .96	

*Note.* Depression refers to either a major depressive disorder or dysthymic disorder. All participants did not have depression at least 1 month prior to baseline as determined using the CIDI. We calculated the risk estimate using the model intercept, the beta coefficients of the predictor variables, the constant (*B*) of CR, and the mean baseline scores of the questionnaires.

LEIDS-RR: Leiden Index of Depression Sensitivity second Revision; NEO-FFI: NEO Five Factor Inventory; IDS-SR: Inventory of Depressive Symptomatology - Self-Report; CIDI: Composite International Diagnostic Interview; yr: year.

## Discussion

Our study showed that cognitive reactivity (CR) is a moderately strong, independent predictor of depressive episodes, both over a period of 2 years and 9 years. Further inspection, however, showed that CR is a stronger predictor of first depression onset than of relapse. CR may also play a role, albeit smaller, in the second episode of depression. Regardless, a personal history of depression, especially three or more past episodes, is a stronger predictor of future relapse than CR. Relapse is very likely, with higher risks for women than men (>70%).

Comparing 2-year and 9-year risk, results were generally similar: the contribution of CR weakened progressively due to the strong effect of depression history. However, for 9-year depression risk, CR may play a slightly stronger role in 9-year relapse than 2-year relapse in individuals with a history of one or two past depressive episodes. Moreover, neuroticism was an active predictor after the first depressive episode, and neuroticism contributed more to 2-year relapse risk than CR. However, this finding was not consistent for 9-year relapse risk.

Our results confirm that people whose negative cognitions are easily activated by dysphoric mood states (i.e., those with high CR scores) are more likely to transition to a first depressive episode, which is consistent with previous literature (Kruijt et al., 2013). However, previous studies both confirmed (Elgersma et al., 2015; Jarrett et al., 2012; Lethbridge & Allen, 2008; Otto et al., 2007; Perez & Rohan, 2021; van Rijsbergen et al., 2013) and did not confirm (Alloy et al., 2006; Bockting et al., 2015; Figueroa et al., 2015; Furukawa et al., 2021; Kuyken et al., 2010; Segal et al., 2006) that CR predicts relapse. This may be explained by the use of a different questionnaire to measure CR (i.e., Dysfunctional Attitudes Scale) (Weissman, 1979). The DAS uses a mood induction procedure and two parallel forms (pre- and post-procedure) to calculate a change score, which reflects CR (Weissman, 1979). The LEIDS-RR, on the other hand, does not make use of a mood-induction procedure, which can be unreliable. Instead, participants are instructed to imagine or recall a mildly sad mood state and then answer conditionally worded items relating to the sad mood (e.g., ‘*When I feel down,* I am more bothered by perfectionism’) (Solis et al., 2017). Moreover, our overall risk estimates for depression relapse were well in line with previous research (Eaton et al., 2008; Solomon et al., 2000). Considering the high contribution of past episodes, especially after three, to relapse risk (> 70%), it may reasonable that CR or any other vulnerability factor could not improve the prediction model and a ceiling effect occurred.

### Clinical and research considerations

After successive depressive episodes, CR appears to play a smaller role in the development of future depression (Lewinsohn et al., 1981). Instead, negative cognitions may become more ingrained in a person’s personality with each successive episode, and the threshold to triggering negative cognitions may become so low that they are sensitive to any stressor or negative mood (Elgersma et al., 2013; van Rijsbergen et al., 2015). Alternatively, negative cognitions may already be embedded in a person’s personality early in life, considering negative cognitions may have developed in childhood, and the threshold to triggering negative cognitions may already be low. When focusing on PDE, we may have, thus, revealed a group of persons with a vulnerable personality structure that may have already been present early in life (Rosenman & Rodgers, 2006), with a higher stress/mood sensitivity (Fox & Moore, 2021) (i.e., neuroticism). The higher stress/mood sensitivity makes it difficult to ascertain at which point negative mood may have activated negative cognitions (i.e., CR). Thus, when looking at 2-year relapse risk, neuroticism scores remained high - or may have already been high, but were simply now selected in our sample.

Additionally, our high relapse-risk estimates suggest a severe risk for chronicity, or the persistence of depressive symptoms over a 2-year period. Past research has shown that neuroticism may be a proxy for chronic depression (Riso et al., 2002; van Eeden et al., 2019), suggesting that some participants may have experienced baseline chronic depression or may have experienced chronic depression at follow-up (van Eeden et al., 2019). Future research may examine CR and chronic episodes. Our findings confirm the importance of early treatment and relapse prevention in clinical treatment for patients with a depression history.

### Strengths and limitations

This was the first study to examine the prediction of depressive episode (both onset and relapse) using the LEIDS-RR and other relevant vulnerability factors, over a longer period of 9 years. We used data from NESDA, a large longitudinal cohort study in diverse settings, which increased generalisability. Finally, we also examined the strength of CR as a predictor, using an analysis that allows for straightforward clinical interpretation.

This study also has limitations. First, while we used the broadest observational period available in NESDA (i.e., CIDI assesses the past year), we may have still missed depressive episodes in the 2-year assessment, thus underestimating depression outcome. Second, calculated weight-scores are only approximations of predictive risk estimates. This is because they use categories of raw scores rather than the continuous values, which sacrifices some information in the model (Bonnett et al., 2019). However, Sullivan et al. (2004) tested the results using the points system and direct functions, and interclass correlations were higher than .90, reflecting high agreement between the two methods. Third, we calculated risk estimates for average baseline profiles. These require replication in a separate, independent sample before they can be used to determine risk in other populations. Fourth, our sample included participants in remission for at least 1 month, and potential residual cognitive symptoms may have affected cognitive reactivity scores in our analyses. This impact, however, is expected to have been minimal, considering that the majority of remitted-depressed participants (*n* = 518) in our sample were remitted for at least 12 months, and 91 were remitted for at least 6 months. Post hoc Cox analysis showed hazard ratios were highly similar and the selection of predictor variables remained the same, when comparing results using a sample without more recent 1-month remitted-depressed participants to our original sample results. Fifth, specific data on antidepressant use or psychological therapies were not collected in NESDA. However, based on post hoc results of a study by Elgersma et al. (2015), which used NESDA data, antidepressant use was not expected to impact CR scores. Regardless, future research could confirm how antidepressants may impact results with CR when including additional factors of neuroticism, residual depressive symptoms, and past depressive episodes. Additionally, without specific data on psychotherapies that change CR (e.g., cognitive therapy, mindfulness therapy) (Cladder-Micus et al., 2018; Figueroa et al., 2015), it was not possible to test for any confounding or mediating effects of psychological treatment in the current study.

## Conclusions

CR is a cogent predictor of first depressive episodes across 2 and 9 years. After the first episode, the influence of CR weakens, and depression history remains the stronger predictor. Neuroticism may also be a moderate predictor of 2-year relapse, but not 9-year relapse, and may be indicative of underlying chronicity. Our results reflect the need for relapse prevention and early treatment of depression to prevent an often recurrent or chronic course of depression.

## Supplementary Material

Supplemental Material

## Data Availability

According to European law (GDPR), data containing potentially identifying or sensitive patient information are restricted; our data involving clinical participants are not freely available in a public repository. However, data are—under some specifications - available upon request *via* the NESDA Data Access Committee (nesda@amsterdamumc.nl). See also our website: www.nesda.nl
