# Supplementary material for: Cognitive reactivity compared to other risk factors in the prediction of depressive episodes over two and nine years: a longitudinal cohort study
Source: Int J Psychiatry Clin Pract. 2025 Mar 16;29(1):32–40. doi: 10.1080/13651501.2025.2476509 (PMC12011020; doi:10.1080/13651501.2025.2476509)
Supplement: Supplemental Material [file IJPC_A_2476509_SM0765.pdf]

SUPPLEMENTARY MATERIAL

Calculation of weight-points and risk estimates

To calculate weight-points, we created categories of the raw questionnaire scores of the predictors (e.g., LEIDS-RR), and we assigned points to each category (Sullivan, Massaro, & D'Agostino, 2004). Specifically, raw questionnaire scores were grouped into categories (e.g., 25-34; 35-44) and assigned representative values (e.g., 25-34 = 30; 35-44 = 40). A reference category was selected to represent the lowest predictive weight. The reference value was subtracted from the category value and multiplied by the beta coefficient of the logistic regression model. These values were then multiplied using the beta coefficient of the logistic regression model. This was then divided by the beta of CR, which was selected as the constant, or the number of regression units that correspond to one point.

Weight-points were then summed, resulting in a total points score. To evaluate these total weight-points, we used ROC analysis, considering statistically-significant interactions. Examining sensitivity, specificity, and the Youden Index (*J*) allowed us to select the optimal cut-off score that would reflect a higher risk for a depressive episode. We strived for a cut-off of the risk prediction score representing equal sensitivity and sensitivity, using the highest Youden score, thus reflecting a lower number of false positives and false negatives (Carter et al., 2016). If the event is less common in the total sample, priority would be given to higher sensitivity, resulting in potentially higher false positives (Carter et al., 2016). Using the optimal cut-off score, we then examined the percentage of correct and false positives in crosstables.

Finally, we calculated baseline risk estimate profiles of men and women using their mean baseline questionnaire scores, the intercept, beta coefficients multiplied by the individual's value for each risk factor, and the weight-point total multiplied by the selected constant (*B*). To calculate the risk estimate, we used the constant (*B*) of CR. We used this formula: *Risk estimate* = 1 / (1 + exp ($-\sum_{i=0} \beta_i X_i$) (Sullivan et al., 2004). Specifically, for 0 episodes: 1 / (1+ exp (-(-5.361) + (CR score *0.038) + (Neuro score*0.029) + (SDS score*0.06) + (Female(1) * .297) + (TOTAL POINTS * 0.038)). For 1-2 episodes = 1 / (1+ exp (-(-5.361) + (CR score *(0.038-0.023)) +(Neuro score*0.029) + (SDS score*(0.06-0.005)) + (Female(1) * .297) + (1.441) + (TOTAL POINTS * 0.038). For ≥ 3 episodes = 1 / (1+ exp (-(-5,361) + (CR score *(0.038-0.039)) + (Neuro score *0.029) + (SDS score *(0.06-0.058)) + (Female(1) * .297) + (3.168) + (TOTAL POINTS *0.038).

Results Regarding Supplementary Tables

Supplementary Table 1 shows an overview of the weight-points of CR and the selected predictors. Supplementary Table 2 shows the distribution of participants based on weight-score after conducting ROC analysis. The ROC analysis demonstrated that a total weight-point score ≥ 78 reflected a sensitivity of .81 and specificity of .66. For 63% (*n* = 1,060) of patients with high scores (≥ 78), 33.5% were accurately predicted to experience depressive episode after two years, at a base rate of 17.6%. There were more than 50% false positives.

Supplementary Table 4 shows the risk for developing nine-year depression based on the Cox analysis and subgroups. In those with 1-2 PDE, CR was statistically significant in the base model (C), with a hazard ratio of 1.03, 95% CI (1.02-1.04), and only SDS was statistically significant in the adjusted model (D), with a hazard ratio of 1.04, 95% CI (1.02-1.06). In those with ≥ 3 PDE, all predictors were not statistically significant in both the base and adjusted models.

Supplementary Table 1. Overview of logistic regression coefficients and calculation of weight-scores concerning two- and nine-year depression risk (based on Sullivan, Massaro, & D'Agostino, 2004).

			Main effect or 0 past depressive episodes					1-2 past depressive episodes					≥3 past depressive episodes				
Variables	Raw categories	Category values (W_i)	Beta, (β_i)	Pre-points	Converted†	**Final Points 2-yr**	**Final Points 9-yr**	Beta, (β_i)	Pre-points	Converted†	**Final Points 2-yr**	**Final Points 9-yr**	Beta, (β_i)	Pre-points	Converted †	**Final Points 2-yr**	**Final Points 9-yr**
CR	< 12 (REF)	**5 = W_1**	.038 (2-yr) .024 (9-yr)	0	0	**0**	**0**	-.023 (2-yr) -.005 (9-yr)	0	0	**0**	**0**	-.039 (2-yr) -.022 (9-yr)	0	0	**0**	**0**
	12-22	18		.49	13.00	**13**	**13**		.19	5.13	**5**	**10**		-.01	-.34	**0**	**1**
	23-35	29		.91	24.00	**24**	**24**		.36	9.47	**9**	**19**		-.02	-.63	**-1**	**2**
	36-52	44		1.48	39.00	**39**	**39**		.58	15.39	**15**	**31**		-.04	-1.03	**-1**	**3**
	53-66	60		2.09	55.00	**55**	**55**		.82	21.71	**22**	**43**		-.05	-1.45	**-1**	**5**
	> 67	79		2.81	74.00	**74**	**74**		1.11	29.21	**29**	**59**		-.07	-1.95	**-2**	**6**
Gender	Male (REF)	**0 = W_2**	.297 (2-yr) .204 (9-yr)	0	0	**0**	**0**	N.S.					N.S.				
	Female	1		.30	7.82	**8**	**9**										
Neuro	< 24	**20 = W_3**	.029 (2-yr) .016 (9-yr)	0	0	**0**	**0**	N.S.					N.S.				
	25-34	30		.29	7.63	**8**	**7**										
	35-44	40		.58	15.26	**15**	**13**										
	>45	50		.87	22.89	**23**	**20**										

SDS	< 10<br>10-19<br>20-29<br>30-39<br>>40	**5 = W_4**<br>15<br>25<br>35<br>45	.060 (2-yr)<br>.066 (9-yr)	0<br>.60<br>1.20<br>1.80<br>2.40	0<br>15.79<br>31.58<br>47.37<br>63.16	**0**<br>**16**<br>**32**<br>**47**<br>**63**	**0**<br>**27**<br>**55**<br>**82**<br>**110**	-.005 (2-yr)<br>-.029 (9-yr)	0<br>.55<br>1.10<br>1.65<br>2.20	0<br>14.47<br>28.95<br>43.42<br>57.89	**0**<br>**14**<br>**29**<br>**43**<br>**58**	**0**<br>**15**<br>**31**<br>**46**<br>**62**	-.058 (2-yr)<br>-.040 (9-yr)	0<br>.02<br>.04<br>.06<br>.08	0<br>.53<br>1.05<br>1.58<br>2.10	**0**<br>**0**<br>**1**<br>**2**<br>**2**	**0**<br>**11**<br>**22**<br>**32**<br>**43**
1-2 episodes	0 (REF)<br>1-2	**0 = W_5**<br>**1**	1.441 (2-yr)<br>1.50 (9-yr)	0<br>1.441	0<br>37.92	**0**<br>**38**	**0**<br>**62**										
≥3 episodes	0 (REF)<br>>2	**0 = W_6**<br>**1**	3.168 (2-yr)<br>2.621 (9-yr)	0<br>3.168	0<br>83.37	**0**<br>**83**	**0**<br>**109**										

† The constant (*B*) used to calculate the points is *B* = .038 (the Beta for Cognitive Reactivity).
[a]The beta coefficient for the interaction of the variables with 0 episodes is .00. This makes the results equivalent to the main effect of the variable.
Abbrev. CR = cognitive reactivity; Neuro = neuroticism; SDS = subclinical depressive symptoms; REF = Reference; N.S. = Not selected; Round points = Rounded points; yr = year.
Note. **Variables** = Risk factors included in the risk prediction model; **Beta (β)** = The regression coefficients of the logistic regression corresponding to the variables (see Table 2); **Reference (W_i)** = The reference value for each category was determined; for the IDS-SR and NEO-FFI scores we used the midpoint of each category (range 1st to 99th percentile), which were based on quartiles within the dataset. Furthermore, we determined the base category for each variable, for the reference ($_{REF}$) profile, with the lowest expected risk. **Pre-points =** Calculated as "Beta * (W_i-W_{iREF})." We determined how far each category is from the base category in regression units (W_i-W_{iREF}); multiplying the beta by the difference between reference value for the specific category and the reference value for the base category. Pre-points of 9-year depression not shown. **Converted (Points) =** Calculated as "Beta * (W_i-W_{iREF})/ B." In order to compute the risk prediction points, we had to set a constant B for the point system, or the number of regression units that will correspond to one point. We calculated the converted points by dividing the Pre-points by the constant. Converted points of 9-year depression not shown. **Final points =** Points were rounded to the nearest integer. **Interactions with past depressive episodes** were not statistically significant for the variables of Gender and Neuroticism, *p* > .05.

Supplementary Table 2. Participants identified with depressive episode after two years based on low or high total weight-scores of the selected predictors.

**Total weight-score**[a]	**Participants with a depressive episode**		**Total participants**
	No	Yes	
Low: 0-77	914<br>Spec: 0.81<br>NPV: 94.2%	56<br>-<br>False neg: 5.8%	629
High: 78-168	478<br>-<br>False pos: 66.5%	241<br>Sens: 0.66<br>PPV: 33.5%	1060
**Total participants (%)**	1392 (82.4%)	297 (17.6%)	1689 (100%)

Note. For this analysis, the total sample was used, *N* = 1689; the base rate was 17.6%. In the sample, the total prediction score ranged from 0 to 156.

[a] Low and high scores were based on ROC analysis, with approximately equal sensitivity and specificity and highest Youden score of .47.

Abbrev. Spec = Specificity; NPV = Negative predictive value; Sens = Sensitivity; False neg = False negative; PPV = Positive predictive value; ROC = Receiver operating characteristics.

Supplementary Table 3. Prediction of depressive episodes based on cognitive reactivity across nine-years, adjusted for other relevant baseline factors, using Cox's proportional hazards analysis, examining subgroups of depression history.

**Sample:**	**0 Past Episodes (*n* = 891)**		**1-2 Past Episodes (*n* = 551)**		**≥ 3 Past Episodes (*n* = 292)**	
**Outcome:**	**First Episode**		**Next Episode - Relapse**		**Next Episode - Relapse**	
**Predictors**	(A) Base: HR (95% CI), *p*-value	(B) Adjusted: HR (95% CI), *p*-value	(C) Base: HR (95% CI), *p*-value	(D) Adjusted: HR (95% CI), *p*-value	(E) Base: HR (95% CI), *p*-value	(F) Adjusted: HR (95% CI), *p*-value
Cognitive reactivity	1.05 (1.04-1.06), <.001*	1.03 (1.02-1.04), <.001*	1.03 (1.02-1.04), <.001*	1.01 (1.00-1.02), .16	1.01 (1.01-1.02), .174	1.00 (.99-1.01), .87
Age	N.I.	.99 (.97-1.00), .06§	N.I.	1.00 (.99-1.01), .53	N.I.	1.00 (.98-1.01), .67
Female[a]	N.I.	1.73 (1.12-2.68), .01*	N.I.	.89 (.65-1.20), .43	N.I.	1.36 (.90-2.05), .14
Education	N.I.	.94 (.89-1.00), .04*	N.I.	.95 (.95-1.05), .95	N.I.	.98 (.92-1.03), .39
Neuroticism	N.I.	.99 (.96-1.03), .77	N.I.	1.02 (.99-1.04), .23	N.I.	1.02 (.99-1.06), .21
SDS	N.I.	1.07 (1.04-1.10), <.001*	N.I.	1.04 (1.02-1.06), <.001*	N.I.	1.01 (.99-1.03), .290

* $p < .05$
[a] "Male gender" was considered the reference category.
[b] "0 past episodes" was considered the reference category.
Abbrev. HR = Hazard ratio; CI = Confidence interval; N.I. = Not included; SDS = Subclinical depressive symptoms.
